# The Construction and Comprehensive Analysis of Inflammation-Related ceRNA Networks and Tissue-Infiltrating Immune Cells in Ulcerative Colitis Progression

**DOI:** 10.1155/2021/6633442

**Published:** 2021-07-06

**Authors:** Jia-Wei Lu, Aimaier Rouzigu, Li-Hong Teng, Wei-Li Liu

**Affiliations:** ^1^Department of Gastroenterology, Sir Run Run Shaw Hospital, School of Medicine, Zhejiang University, Hangzhou, 310016 Zhejiang Province, China; ^2^Department of General Practice, Sir Run Run Shaw Hospital, School of Medicine, Zhejiang University, Hangzhou, 310016 Zhejiang Province, China

## Abstract

Ulcerative colitis (UC) is a common disease with great variability in severity, with a high recurrence rate and heavy disease burden. In recent years, the different biological functions of competing endogenous RNA (ceRNA) networks of long noncoding RNAs (lncRNAs) and microRNAs (miRs) have aroused wide concerns, the ceRNA network of ulcerative colitis (UC) may have potential research value, and these expressed noncoding RNAs may be involved in the molecular basis of inflammation recurrence and progression. This study analyzed 490 colon samples associated with UC from 4 gene expression microarrays from the GEO database and identified gene modules by weighted correlation network analysis (WGCNA). CIBERSORT detected tissue-infiltrating leukocyte profiling by deconvolution of microarray data. LncBase and multiMIR were used to identify lncRNA-miRNA-mRNA interaction. We constructed a ceRNA network which includes 4 lncRNAs (SH3BP5-AS1, MIR4435-2HG, ENTPD1-AS1, and AC007750.1), 5 miRNAs (miR-141-3p, miR-191-5p, miR-192-5p, miR-194-5p, and miR196-5p), and 52 mRNAs. Those genes are involved in interleukin family signals, neutrophil degranulation, adaptive immunity, and cell adhesion pathways. lncRNA MIR4435-2HG is a variable in the decision tree for moderate-to-severe UC diagnostic prediction. Our work identifies potential regulated inflammation-related lncRNA-miRNA-mRNA regulatory axes. The regulatory axes are dysregulated during the deterioration of UC, suggesting that it is a risk factor for UC progression.

## 1. Introduction

Ulcerative colitis (UC) is a chronic and incurable inflammatory disease, which most often affects the gastrointestinal tract. A recent study also reported that UC prevalence is increasing rapidly worldwide with a high recurrence rate and heavy disease burden (1). Patients with UC need a lifelong course of drugs, which results in high psychological and financial burdens to patients. An emerging biological agent, mainly monoclonal antibodies against cytokines, has been developed, including anti-TNF-*α* agent (infliximab, golimumab), integrin antagonists (vedolizumab)and janus kinase inhibitor (tofacitinib) (2). Nevertheless, up to 30% of patients show no clinical benefit following biopharmaceutical. It is urgent to deepen the understanding of the pathogenesis of ulcerative colitis progression at the molecular level to determine new therapeutic and disease surveillance strategies. The gene expression network of the disease has been predicted to be sophisticated and fathomable, involving a large variety of characters, such as transcription factors (TFs), microRNAs (miRNAs), long noncoding RNAs (lncRNAs), and protein-coding genes (mRNAs).

One of the regulated axes is called the competing endogenous RNA network (ceRNA), and the ceRNA hypothesis was first proposed by Salmena et al. in 2011 (3). LncRNA competes with mRNA for miRNA by acting as a miRNA sponge, and increases the downstream mRNA expression.

The role of the ceRNA network has attracted much attention in recent years. Recently, there has been a lot of research and analysis of ceRNA, of which tumor analysis is the most extensive. Many studies have confirmed that lncRNAs are mediated by proliferation, metastasis, drug sensitivity, and tumor progression. Recent research expanded its function into the field of nontumor. Few studies on ceRNAs have focused on IBD-related mechanisms. So far, Nie and Zhao et al. have shown that Lnc-ITSN1-2 promotes Th1/Th17 cell differentiation and CD4+ T cell activation by sponging miR-125a in increased IBD develops inflammatory cytokines of IBD (4). Ye et al. have shown that the dysregulation of circRNA_103516 in PBMCs may participate in IBD through hsa-miR-19b-1-5p sponging (5).

The complex network of lncRNA-miRNA-mRNA regulatory mechanisms is difficult to determine by exploring individual pair interactions. Therefore, high-throughput sequencing of RNA isolated by crosslinking immunoprecipitation techniques (HITS-CLIP) can directly identify multiple targeting sequences in the samples (6). The high-throughput experimental data provide molecular interaction prediction data (7).

This research emphasizes establishing a regulatory network rather than analyzing individual genes that focus on a specific molecular interaction. The functions of the characteristic RNAs were investigated by exploring multiple UC-related microarrays from Gene Expression Omnibus (GEO) datasets. We elucidated their possible participation in UC pathogenesis and established an ulcerative colitis progression-associated ceRNA network. Overall, the effects and potential underlying molecular mechanisms of RNAs on the pathological process of ulcerative colitis were elaborated through interactions with specific RNAs. We explored an inflammatory-related ceRNA network including 4 lncRNAs, 5 miRNAs, and 52 mRNAs. The ceRNA network may involve the interaction among tissue-infiltrating immune cells, including neutrophils, macrophage M0/M1, CD8+T cell, and regulatory T cell. The analysis indicated that the ceRNA network might become a potential genetic risk factor for ulcerative colitis.

## 2. Materials and Methods

### 2.1. Data Collection and Data Processing

The Gene Expression Omnibus (GEO, https://www.ncbi.nlm.nih.gov/geo/) database was searched for publicly available studies and samples that fulfilled the following criteria for analysis: (1) the species of the samples was Homo sapiens, (2) the gene expression data series contained UC colon tissue and normal colon tissue samples, (3) the selected dataset should be over 15 samples, and (4) the characteristic of samples from a gene expression study contained UC activity assessment. We used five adult human UC microarray datasets from GSE48959, GSE73661, GSE75214, GSE87466, and GSE92415. Among them, GSE48959, GSE73661, and GSE75214 were hybridized to Affymetrix Human Genome Affy Human Gene 1.0ST Genechips (Affy Human Gene 1.0ST, Affymetrix), while GSE87466 and GSE92415 were profiled using U133A Genechips (HG-U133A, Affymetrix); miRNA array profiling from GSE48959 was performed with the Affymetrix Multispecies miRNA-2 Array by reannotating the mRNA microarray data for lncRNAs. Samples contain a gene expression matrix and baseline characteristics of 507 ulcerative colitis samples and 62 normal colon samples, of which 17 colitis samples and 10 normal samples come from miRNA datasets. Microarray data were normalized, and the batch effect was assessed and removed by the removeBatchEffect function from the limma R package.

### 2.2. Established Tissue-Infiltrating Immune Cell Signature Matrix (LM22)

Using CIBERSORT analysis to analyze LM22 abundance in each sample and filter out abnormal data (8), subsequently, we calculated the correlation coefficient between the abundance of the cell subset of the samples and the rank variable of the disease status of their represented samples.

### 2.3. Identify Modules of Coexpressed Genes within Gene Expression Networks

We used weighted gene coexpression network analysis (WGCNA) to identify modules of coexpressed genes within gene expression networks (9). WGCNA was implemented in R with the following major parameters: signed network, power of 20, maxBlockSize of 20000, minModuleSize of 50, verbose of 3, and mergeCutHeight of 0.25. Module membership (MM) represents the intramodular connectivity of any gene in a given module. A higher absolute value of MM represented that a gene has a greater correlation with the module eigengenes (MEs). The gene significance value (GS) implies the correlation between the clinical features and the gene expression in a module. A higher value of GS indicates the increased biological significance of a gene for a given clinical trait. Hub genes in key modules were identified based on the following thresholds: ∣MM | >0.8 and GS > 0.2.

### 2.4. Weighted Gene Coexpression Network Meta-Analysis

The preservation and reliability of the modules was checked by module preservation analysis. These datasets were independently processed depending on the platform, and the expression of module genes in corresponding datasets was used as input data to measure the degree of preservation in each dataset (10). The following thresholds for *Z*summary were used: no preservation (*Z*summary < 2), weak-to-moderate evidence of preservation (2 < *Z*summary < 10), and strong evidence of module preservation (*Z*summary > 10).

### 2.5. Enrichment Analysis and Enrichment Map of Enrichment Result

To identify represented pathways in coexpression modules related to UC development, we used ClusterProfiler in the R/Bioconductor package (11), Hypergeometrictest calculated by ClusterProfiler was applied to analyze the significantly enriched GO terms. Results were visualized by bar plots created by the ClusterProfiler package. The Enrichment Map results from gene set enrichment analysis created by the enrichplot package (R package).

### 2.6. Protein-Protein Interaction (PPI) Networks

StringApp constructed a comprehensive human PPI network (12). This study integrates hub genes of the differential coexpression module. A PPI network was constructed by mapping the gene list to the PPI network. We overlapped our gene list with the PPI network, set the confident threshold of 0.7, and removed the noninteracting nodes.

### 2.7. Construction of a ceRNA Network

Based on the hypothesis, the candidate lncRNA and mRNA expression must have the same variation trend, while miRNA should have the opposite trend. lncRNA-miRNA interactomes were performed using the LncBase experimental database (13), and miRNA-mRNA interactions were obtained from the R package “multiMIR” (14). The obtained lncRNA-miRNA pairs and miRNA-mRNA pairs were combined to construct a ceRNA network. The Cytoscape software (v3.8.0) was used to visualize the ceRNA networks (15).

### 2.8. Establish a Severity Detection for the UC Patients Using a Decision Tree

The patient's clinical data were extracted from the clinical data of UC patients in two GEO datasets. 328 UC samples studied in GSE73661 and GSE92415 were included. Outliers were filtered out by choosing an appropriate cut parameter of the height of the tree cut in the dendrogram. We established a decision tree model using rpart (R package). We train and provide better model results by using Caret (R package). After performing 10-fold cross-validations, the cost-complexity parameter (Cp) value in which the test error was minimized was selected as the optimal Cp value. Classification models were evaluated based on the area under the ROC curve (AUC) using the “pROC” (16) software package (R package) and confusion matrix (R package) (17).

## 3. Results

### 3.1. Preprocessing of the lncRNA/mRNA Datasets and Construction of Weighted Gene Coexpression Networks

**Figure 1:**
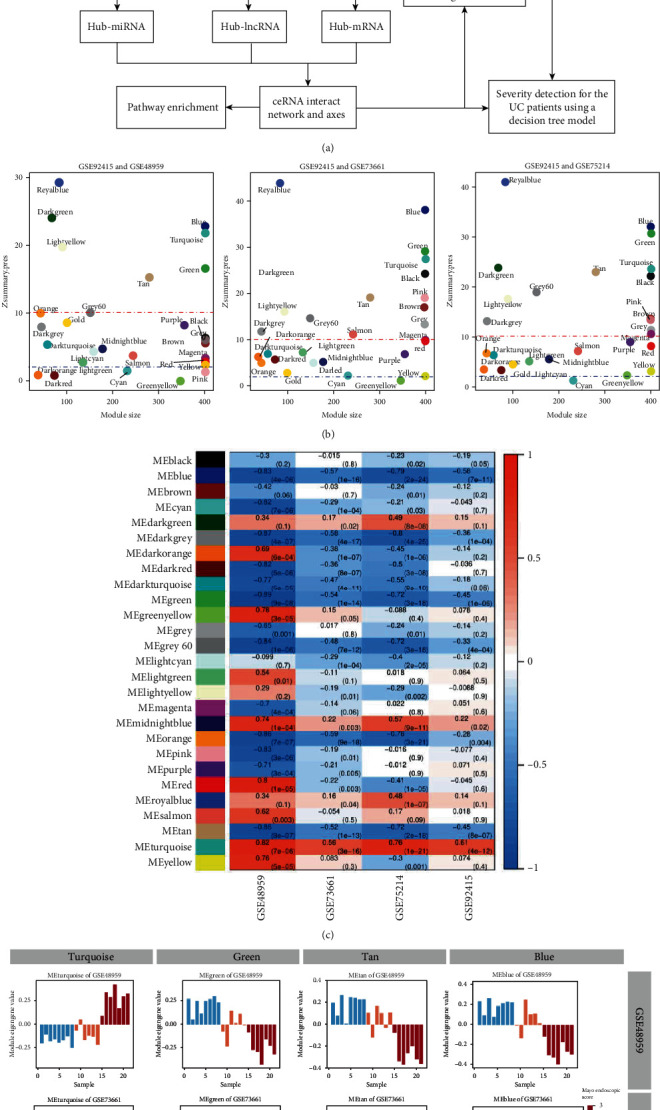
Preprocessing of the lncRNA/mRNA datasets and construction of weighted gene coexpression networks. (a) Flowchart for an overview of the present analysis. (b) Preservation of GSE92415 network modules in different datasets. The *y*-axis displays the *Z* score for each module. Labels beside each module (colored dot) represent the corresponding module in the reference dataset. The *x*-axis represents the number of genes in the module. *Z* scores of less than 2 (blue bottom line) imply no evidence for module preservation, while scores exceeding 10 (red line) imply strong evidence for module preservation. (c) Module trait relationship: a matrix with the module-trait relationships (MTRs) (correlation coefficients) and corresponding *P* values (in brackets) between modules on the *y*-axis and disease progression traits of four datasets on the *x*-axis. (d) The relationship between the module eigengene and disease severity: the *y*-axis represents the module eigengene expression value of the sample, and the samples are sorted from mild to severe disease status.

The overview of this study is shown in Figure 1(a). We screened qualified datasets from GEO, and finally, four gene expression datasets were downloaded from GEO (GSE48959 (18), GSE73661 (19), GSE75214 (20), and GSE92415 (21)). In summary, 52 normal colon biopsy samples and 438 UC colon biopsy samples were included (the total patients' characteristic is shown in Tables S1 and S2) All of these samples contain an evaluation of disease activity by both endoscopy and symptoms. The evaluation variables are further categorized into ordinal variables. Ordinal variables allow us to order the disease severity in terms of which category has less and which category has more severe severity represented by the variable. An ordinal variable is ranked by the Mayo score from GSE92415, Mayo endoscopic score from GSE73661, and disease activity assessment from GSE48959 and GSE75214 (Table S8). We identified 15088 common mRNAs and 1866 common lncRNAs from four datasets. Using the WGCNA method, 25 gene modules were constructed. A gene module is considered a set of coexpressed genes to which the same set of transcription factors binds. Those modules were first validated by assessing their preservation across datasets (Figure 1(b) and Table S4). Remarkably, the coexpression structure of those modules from GSE92415 can be reproducibly identified in either of three independent expression datasets, especially in blue, dark-green, green, grey60, light-yellow, royal blue, tan, and turquoise modules. The next step is to illustrate the correlation between the gene module and disease severity. We noticed that turquoise modules were positively related to the severity of ulcerative colitis among four datasets (Figure 1(c)), green, blue, and tan modules were negatively related to the development of ulcerative colitis among four datasets (Figure 1(c)), and the bar plots revealed that the eigengene value of modules correlated with the disease severity (Figure 1(d)). The above work shows that four modules (turquoise, green, blue, and tan) are significantly related to ulcerative colitis severity. Subsequently, we used a relatively high criterion to select hub RNAs on 4 modules (Table S5). Finally, we identified 329 hub mRNAs and 23 hub lncRNAs in the turquoise module, 189 hub mRNAs and 13 hub lncRNAs in the blue module, 76 hub mRNAs and 1 hub lncRNA in the green module, and 31 hub mRNAs and 3 lncRNAs in the tan module.

### 3.2. Construction of Weighted Gene Coexpression Networks in the miRNA Dataset

**Figure 2:**
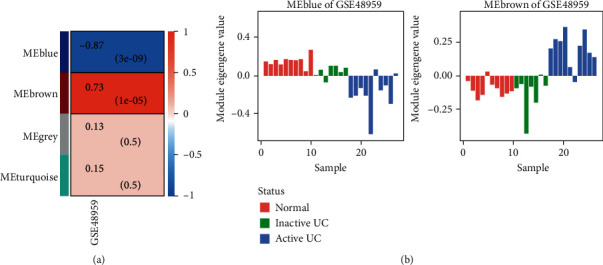
miRNA module-trait relationship. (a) A matrix with the miRNA module-trait relationships (correlation coefficients) and corresponding *P* values (in brackets) between modules on the *y*-axis and disease progression traits of multiple datasets on the *x*-axis. (b) The relationship between the module eigengene and disease severity: the *y*-axis represents the module eigengene expression value of the sample, and the samples are sorted from mild to severe disease status.

The GSE48959 microarray dataset also contains miRNA expression data. In total, there were 8 normal colon biopsy samples and 16 ulcerative colitis colon biopsy samples (the total patients' characteristic is shown in Table S3); we obtained 1067 common miRNAs from the datasets. Using WGCNA, two modules correlated with ulcerative colitis progression are detected (Figure 2(a)), of which 11 hub miRNAs of the brown module were upregulated and 20 hub miRNAs of the blue module were downregulated (Table S6), and we noticed that the module eigengene value also correlated with the disease severity (Figure 2(b)).

### 3.3. GO Enrichment and Pathway Analysis of Modules

**Figure 3:**
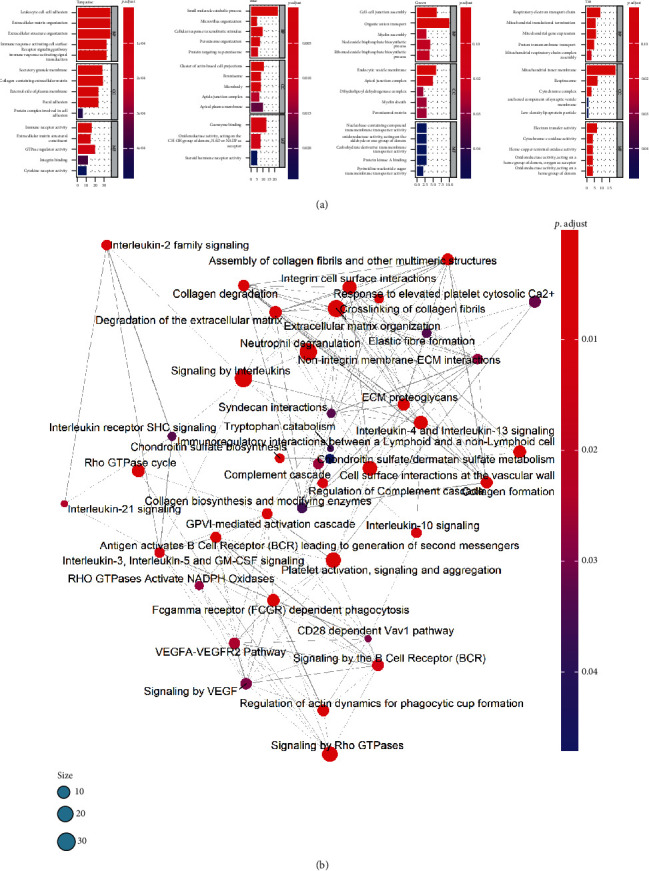
GO enrichment and Reactome pathway analysis for dysregulated gene modules. (a) Bar charts showing the top 5 GO terms for biological process (BP), cellular component (CC), and molecular function (MF) in four modules (*P* < 0.05). (b) Pathway crosstalk among gene-enriched pathways of the turquoise module. Nodes represent pathways, and edges represent crosstalk between pathways.

We performed pathway and Gene Ontology (GO) enrichment analyses of the identified hub gene set function in modules. The main functions and pathways enriched by the hubs in these modules are shown. The function enrichment of the turquoise module was found to participate in inflammation development (*P* < 0.05, Figure 3(a)). These genes were enriched for the Gene Ontology categories related to leukocyte cell adhesion and proliferation, as well as neutrophil activation and degranulation, and also stimulated for cytokine release and cytokine receptor activity; pathway enrichment demonstrated that hub genes were involved in inflammation mediated by extracellular matrix (ECM) organization, leukocyte-cell adhesion, immune response-activating cell receptor signal, cytokine receptor activity, etc. (*P* < 0.05, Figure 3(b)). The blue module showed a significant decrease in a small molecular catabolic process, microvillus organization, and steroid hormone receptor activity. The green module showed a significant decrease in cell-cell junction, organic anion transport, and endocytic vesicle membrane formation. The tan modules showed a significant decrease in ion channel/transporter activity, lipid metabolism, and biological oxidation (*P* < 0.05, Figure 3(a)). The results may indicate that mitochondrial function and various metabolisms were dysfunctional during the progression. Tissue structure formation and bowel barrier were also damaged, preventing mucosal wound healing and disrupting the mucus's protective function layer and contributing to recurrent and deterioration of inflammatory stimulation.

### 3.4. Construction of an Inflammation-Related ceRNA Network

**Figure 4:**
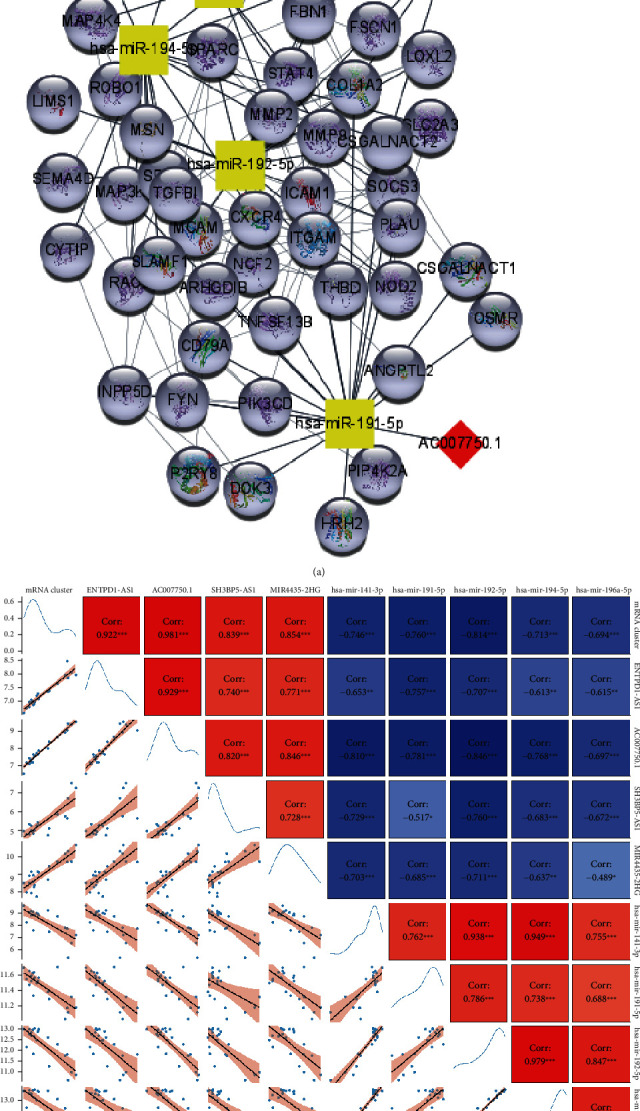
Construction and correlation analysis of the ceRNA regulatory network. (a) lncRNA-miRNA-mRNA interacted network. The squares represent the downregulated miRNA, the rhombus represents the upregulated lncRNA, and the circles represent the upregulated mRNA. (b) The data were visualized by heat map, with a positive correlation in red and a negative correlation in blue. The graduated color represents the correlation coefficient (ranging from 0, pale colors, to ±1, deep colors). Pearson's correlation test calculated the correlation coefficient.

We identified the turquoise module as significantly upregulated and mainly related to the inflammation process; then, we constructed the PPI network-associated turquoise module's mRNAs (Figure S1). Based on the ceRNA hypothesis, we selected the negative interactomes from the ceRNA network to construct regulatory axes. 23 upregulated hub lncRNAs from the turquoise lncRNA/mRNA module, 20 downregulated miRNAs from the blue miRNA module, and 195 upregulated mRNAs in the PPI network from the turquoise lncRNA/mRNA module were first screened. The negatively correlated miRNA-mRNA pairs in the ceRNA network were detected through synthesizing miRDB, miRTarBase, and TargetScan validation, and lncRNA-miRNA pairs were detected by using the DIANA-LncBase v3 database (see Section 2.7). Finally, a network consisting of 4 lncRNAs, 5 miRNAs, and 52 mRNAs was constructed and visualized (Table 1 and Figure 4(a)). A diagram of the correlation relationships between lncRNAs, miRNAs, and mRNAs revealed that all miRNA expressions significantly correlated with their targeted lncRNAs and targeted mRNAs in GSE48959 datasets (Figure 4(b)).

### 3.5. The Correlation of the Disease Progression and the Abundance of Tissue-Infiltrating Immune Cells

**Figure 5:**
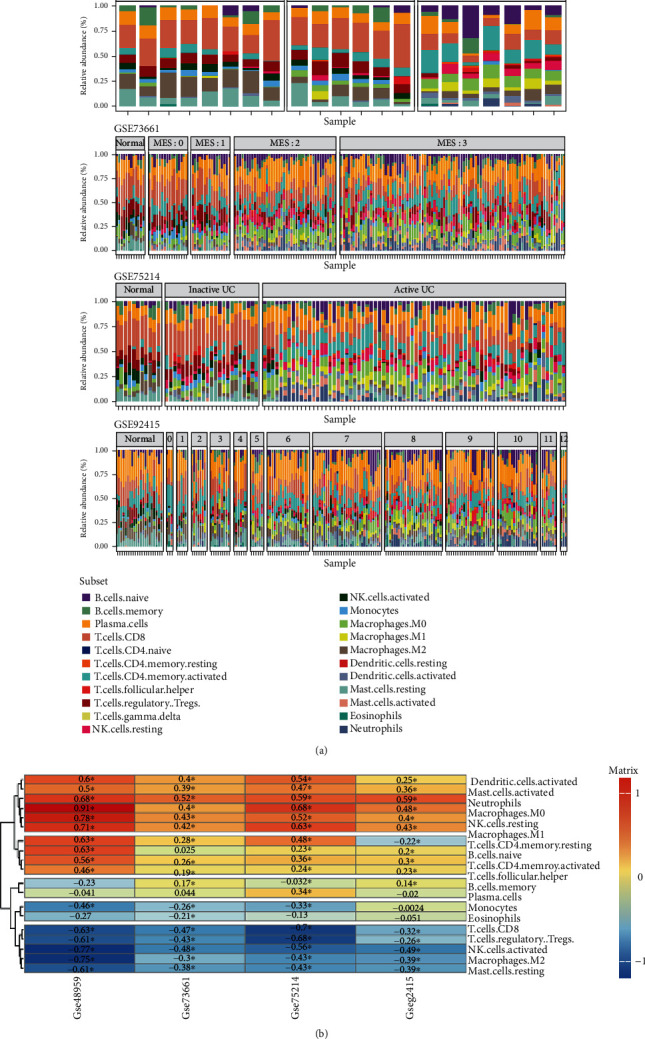
The correlation of disease progression and changes in tissue-infiltrating immune cells. (a) Enrichment scores for 22 tissue-infiltrating immune cell subpopulations on four gene expression datasets based on deconvolution by CIBERSORT. The results are listed in order of disease severity assessment (GSE73661, Mayo endoscopic score (MES): 0-3; GSE48959 and GSE75214, disease activity assessment: normal, inactive UC, active UC; GSE92415, Mayo score: 0-12). (b) A heat map of the Spearman rank correlation coefficient between the proportion of 19 tissue-infiltrating immune cells and disease characteristics on four gene expression datasets. Asterisks denote significant correlations after *P* value corrections (*P* < 0.05).

We discuss the relative abundance of different tissue-infiltrating immune cell types analyzed by CIBERSORT. The results are listed in order of severity for the disease (Figure 5(a)). As the disease progressed, immune cell composition changes as an evolution of the disease. For better understanding, we analyze Spearman's rank correlations between the abundance of tissue-infiltrating immune cells and severity for the disease (Figure 5(b)), and the abundance of six subsets of immune cells (neutrophils, resting NK cell, macrophage M0/M1, activated dendritic cell, and activated mast cell) was correlated positively and significantly with the progression. In comparison, five subsets (regulatory T cell, CD8+ T cell, activated NK cell, macrophage M2, and resting mast cell) were significantly negatively correlated. In summary, the results showed that the deterioration of UC is correlated with the infiltration of the proinflammatory cell.

### 3.6. The Relationship between the ceRNA Network and Inflammation

**Figure 6:**
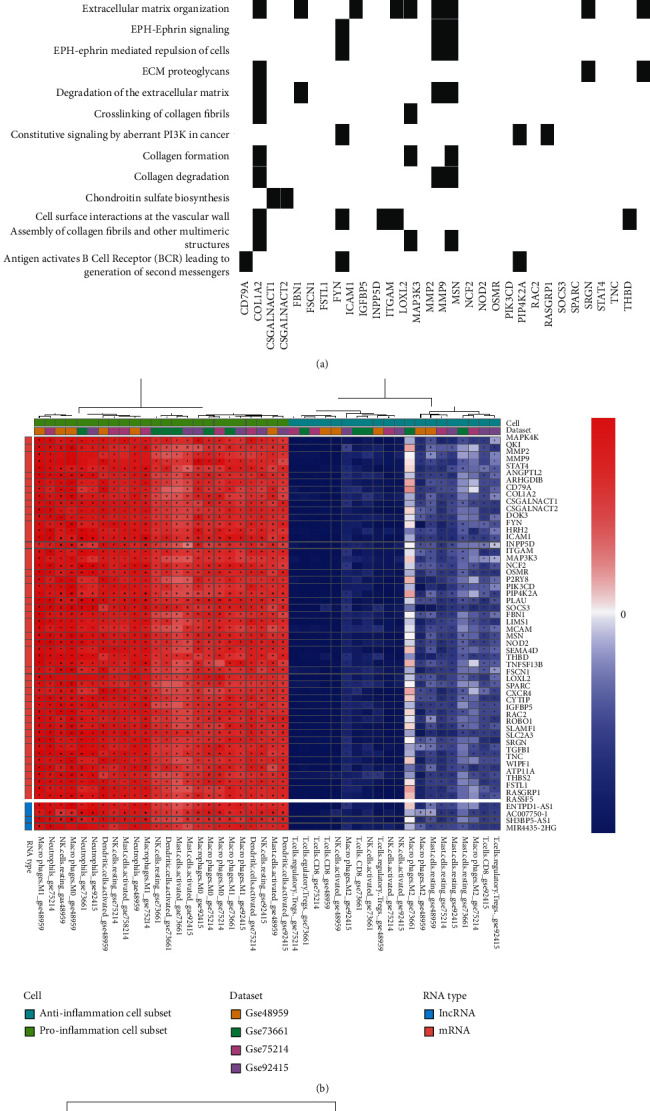
Correlation between the gene of the ceRNA network and inflammatory pathway. (a) Heat map represents an association matrix of mRNAs in the ceRNA network and Reactome pathway terms. (b) Correlation analysis between the abundance of tissue-infiltrating immune cells and the expression of lncRNAs/mRNAs in respective datasets. Asterisks denote significant correlations after *P* value corrections (*P* < 0.05). (c) Correlation analysis between the abundance of tissue-infiltrating immune cells and the expression of miRNAs in respective datasets. Asterisks denote significant correlations after *P* value corrections (*P* < 0.05).

The enrichment showed that the ceRNA network genes were mainly enriched in interleukin family signals (especially in IL-4 and IL-13 signaling), neutrophil degranulation, adaptive immunity, cell surface interaction at the vascular wall, and integrin-cell surface interactions (Figure 6(a)). We also assessed the correlation between these expressions of RNAs and tissue-infiltrating immune cells, and the correlation analysis showed that mRNAs, miRNAs, and lncRNAs were moderately and highly associated with most of the UC-related infiltrating immune cells in respective datasets, in particular neutrophil, macrophage M1, macrophage M0, CD8+ T cell, and regulatory T cell (Figures 6(b) and 6(c)).

### 3.7. Establish a Severity Detection for the UC Patients Using a Decision Tree

**Figure 7:**
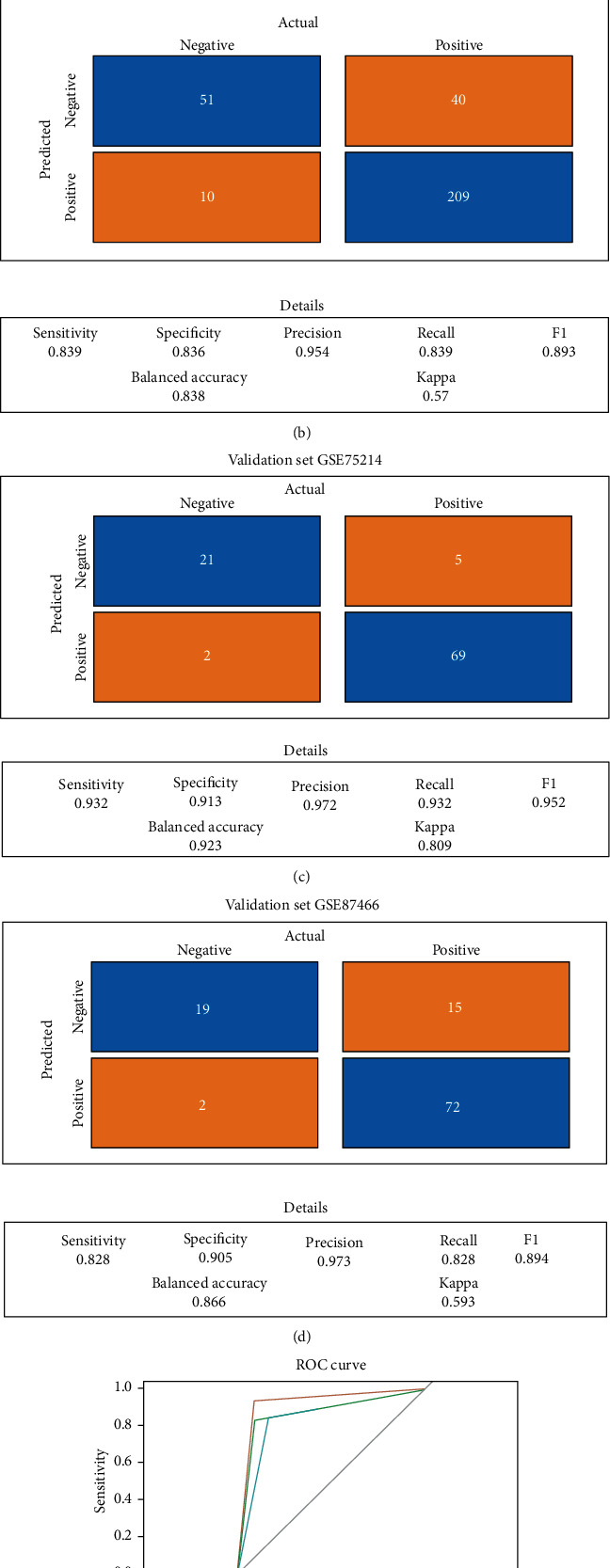
Establishment of a severity detection for the UC patients using a decision tree. (a) Decision tree (the normalized MIR4435-2HG expression values are shown, and all gene expressions were standardized during the calculation). (b) Confusion matrix for the training dataset. (c) Confusion matrix for the validation set GSE75214. (d) Confusion matrix for the validation set GSE87466. (e) ROC curve of the model for the training set and two validation sets. The vertical axis represents sensitivity, and the horizontal axis represents specificity.

We established a severity detection model for detecting disease status based on the gene expression of the microarray sample and verified the robustness of our model predictions and inference to assess their suitability for disease surveillance. Based on the current treatment options for UC, the therapy plan of moderate-to-severe status is quite similar (22) (moderate-to-severe ulcerative colitis defined as having a total Mayo score of 6 to 12 points and Mayo endoscopic score of 2 to 3). Moreover, patients with inactive and mild UC are considered to be in remission status. Therefore, we classified clinical phenotypes into inactive-to-mild or moderate-severe ulcerative colitis, and the normal control group was excluded from this model; then, the expressions of lncRNA in the ceRNA network were treated as diagnosis variables. The 328 UC samples from GSE73661 and GSE92415 were first included. To avoid overfitting, we also detected and filtered out the outlier samples (Figure S2). In total, 249 moderate-to-severe patients (80.3%) among the 310 colon samples were treated as a training set. GSE75214 and GSE87466 (23) were regarded as the external validation set. The decision tree model was fitted by the machine learning method for acquiring the optimized complexity parameter value (Figure 7(a)). Overall, lncRNA MIR4435-2HG was selected as the key feature, with a sensitivity of 83.9%, specificity of 83.6%, and balanced accuracy of 83.8% (Figure 7(b)). There was also a good concordance with validation sets GSE75214 and GSE87466 judged by the model (GSE75214: sensitivity: 93.2%, specificity: 91.3%, and balanced accuracy: 92.3% and GSE87466: sensitivity: 82.6%, specificity: 90.5%, and balanced accuracy: 86.6%, respectively). These are under the ROC in the training set, validation set GSE75214, and validation set GSE87466 which were 0.84 (95% CI 0.79-0.89), 0.92 (95% CI 0.86-0.99), and 0.87 (95% CI 0.79-0.94), respectively, which indicates a good ability to distinguish moderate-to-severe UC (Figure 7(e)). The results reveal that this model may provide a gene expression-based disease surveillance of UC.

## 4. Discussion

In this article, we found that interleukin family-related pathways, focal adhesion, and extracellular matrix adhesion enriched in ceRNA networks are known to play an essential role in ulcerative colitis in the literature review (24–26). Interleukin-13 and interleukin-4 are produced by CD4+ Th2 cells, mediating UC through the shared type II interleukin-4 receptor, as it cooperated with tumor necrosis factor-alpha (TNF-*α*) to regulate the expression of genes responsible for the development of tight junction enteroepithelial cells (27). Verstockt et al. suggested that IL-13R*α*2 on epithelial cells contributes to IBD pathology by negatively regulating goblet cell recovery and epithelial restoration after injury (28). Blocking IL-13R*α*2 might be a promising target for the IBD therapy, safety data of drugs targeting the IL-13 and IL-4 pathway are reassuring, and there were also a small number of studies that demonstrated protective effects of IL-4 pathway inhibition,IL-4 pathways inhibitionrepressed the proliferation of malignant cells and increased apoptosis in a mouse model of colorectal cancer. However, several lines of evidence challenge this safety; Braddock et al. have proven that IL-13 and IL-4 may have roles in the development of colorectal cancers (29). Therefore, more research is needed to eliminate the current disarray in the literature.

We also discovered that the abundance of inflammatory cells in M0/M1 cells, activated mast cells, neutrophils, and cd4t cells was significantly positively correlated with the disease progression. M0/M1 cells are highly related to the deterioration of the disease. Many articles have shown that M1 cells in inflammatory bowel disease are the main cell type that releases inflammatory factors (30–32). It is known that LPS and IFN-*γ* can activate M1 macrophages through the nuclear factor kappa-B (NF-*κ*B) signaling pathway, producing the proinflammatory factors IL-1*β*, IL-6, IL-23, TNF-*α* and ROS (33). Thus, M1 macrophages may be the major inflammatory cell in the early stage of inflammation. The potential function of mast cells to IBD has been demonstrated in experimental studies. Albert-Bayo et al.' reviews conclude that mast cells play a role in intestinal permeability, initiation and maintenance of inflammatory processes with ensuing tissue remodeling and neuropathological stress (34). Combined with the functional enrichment of those three downregulated modules, the tissue lipid/small molecular metabolism level was decreased and tissue and cell structures were also damaged. It was exacerbating the defective gut barrier in ulcerative colitis patients. A proinflammatory cytokine loop overrides anti-inflammatory signals and causes chronic intestinal inflammation.

Based on the results, a potential ceRNA regulatory axis fitted well with the ceRNA pattern, and the lncRNA MIR4435-2HG is highly enriched with several cytokine pathways. The massive report indicated that lncRNA MIR4435-2HG contributes to colorectal cancer development and predicts poor prognosi. MIR4435-2HG was identified as a miRNA sponge for TGF-*β*1 and thus activated TGF-*β* signaling, which indicates that MIR4435-2HG may also play an inflammation-mediated role. In Dong et al., MIR4435-2HG was highly expressed in CRC tissue compared to normal tissues, displaying poor prognosis (35, 36). Overall, the literature review suggested that MIR4435-2HG knockdown could suppress CRC cell proliferation, invasion, and migration. MIR4435-2HG may be a key mediator of both inflammatory processes and colorectal cancer generation. In addition to lncRNAs, miRNAs, as a widely discussed noncoding RNA, are critical in the ceRNA hypothesis. We found a complex regulatory network for the above 5 miRNAs, and the miRNAs have extensively been studied. Mechanisms involving miRNAs have been shown to take part in various autoimmune diseases, including IBD. Wu et al. first evaluated the abnormal expression of miRNA miR-192 in the intestinal tissue of UC patients and decrease TNF-*α*-induced MIP-2-*α* expression, also shown to be profibrotic (37); miR-192 can inhibit the expression of NOD2, inhibiting innate immune system activation via the NF-*κ*B pathway, and inhibit interleukin-8 and CXCL3 messenger RNA expression (38), indicating that mir-192 might protect colon tissue from the damage by participating in the inhibition of innate immune signaling. Another miRNA, mir-194-5p, was confirmed to have the specific differential expression of miR-194 in ulcerative colitis (39), and miR-194 has more attention on several autoimmune diseases. Tian et al. show that overexpression of miR-194 attenuated the release of the proinflammatory cytokine TNF-*α* in PA-activated monocyte THP-1 in rheumatoid arthritis (40). Another downstream miRNA mir-196a is associated with various diseases, including rheumatoid arthritis (41) and colorectal cancer (42–44). In the review about IBD, Ranjha et al. found that miR-196a-2 was also negatively associated with UC (45). However, Brest et al. found that the over expression of miR-196 affected the function of autophagy due to the downregulation of the immunity-related GTPase family M protein (46). The pathogenesis of UC and Crohn's disease is considerably distinct from each other. The dysbiosis and impairment of the epithelial barrier via disruption of tight junctions are strongly implicated in the pathogenesis of UC (22).Studies evaluating biological treatments in patients with severe disease and inadequate response to conventional therapies were remarkably cost-effective. We have attempted to use multiple methods, including the widely used regression model, to establish a diagnostic prediction model, but high-throughput microarray data may not be applicable for constructing the regression model due to big noise and high dimensions. This study tested the performance of supervised machine learning algorithms: Decision Forest Regression (DFR); disease modeling with DFR has several distinct advantages; the approach, which automates the feature selection, efficiently selected the few critical features from the false signal; and the model has a simple structure based on one optimal attribute of each split dot. Our decision tree confirms that lncRNA MIR4435-2HG variables are good predictors. The goodness of fit assessment suggested that our model fits well with the data. The accuracy of the decision tree is also relatively high.

This article addresses several limitations in this literature. First, it is unfeasible for a current database to include all RNA-RNA interaction information. Hence, some of the potential interactomes may not be included in our network, and some critical regulatory RNAs might be lost of the annotations. Second, despite the progress in developing the ceRNA network, the hypothesis still lacks conclusive evidence. Most of the active miRNAs were not readily affected by ceRNA. The natural conditions in cells are difficult to control, and it is easy to overexpress genes artificially, which cannot mimic the normal ceRNA effects in the body.

Moreover, there are not many prediction tools currently available. Third, most of the miRNA-mRNA predicted datasets are based on 3′UTR sequences, which have certain limitations. An accumulating amount of evidence indicates that lncRNAs play an important role in biological functions through multiple regulation levels, which involve transcriptional, posttranscriptional, and epigenetic regulation.

## 5. Conclusions

Our work combined four microarray data from the GEO database and identified potential inflammatory gene-related ceRNA network regulatory axes dysregulated during UC progression. We speculate that the lncRNA-miRNA-mRNA regulatory axes may depend on the interaction among tissue-infiltrating immune cells, including neutrophil, macrophage M0/M1, CD8+ T cell, and regulatory T cell. These RNAs play a crucial role in inflammatory stimulation and its abnormal behavior in UC, as reported. The network and its potential function obtained from the bioinformatics analysis can be examined by future experimental studies.

## Figures and Tables

**Table 1:** ceRNA regulation network of lncRNAs, miRNAs, and mRNAs in UC.

lncRNA	miRNA	mRNA
AC007750.1	hsa-miR-191-5p	ANGPTL2, ARHGDIB, CD79A, COL1A2, CSGALNACT1, CSGALNACT2, DOK3, FYN, HRH2, ICAM1, INPP5D, ITGAM, MAP3K3, NCF2, OSMR, P2RY8, PIK3CD, PIP4K2A, PLAU, SOCS3

SH3BP5-AS1	hsa-miR-192-5p hsa-miR-194-5p hsa-miR-196a-5p	FBN1, LIMS1, MCAM, MSN, NOD2, PLAU, SEMA4D, THBD, TNFSF13B, FSCN1, LOXL2, COL1A2, CSGALNACT2, SPARC, CSGALNACT2, CXCR4, CYTIP, IGFBP5, MAP3K3, MSN, RAC2, ROBO1, SLAMF1, SLC2A3, SOCS3, SRGN, TGFBI, TNC, WIPF1, ATP11A, FSCN1, COL1A2, CSGALNACT2, FBN1, THBS2, FSTL1, RASGRP1, RASSF5

MIR4435-2HG	hsa-miR-196a-5p	ATP11A, FSCN1, COL1A2, CSGALNACT2, FBN1, THBS2, FSTL1, RASGRP1, RASSF5

ENTPD1-AS1	hsa-miR-194-5p hsa-miR-196a-5p	MAP4K4, QKI, MMP2, MMP9, STAT4, ATP11A, FSCN1, COL1A2, CSGALNACT2, FBN1, THBS2, FSTL1, RASGRP1, RASSF5

## Data Availability

Previously reported gene expression microarray data were used to support this study and are available at the Gene Expression Omnibus database (https://www.ncbi.nlm.nih.gov/geo/). These prior studies (and datasets) are cited at relevant places within the text as references [18–21, 23]. The processed data and code are available from the corresponding authors upon request.

## Abbreviations

UC: Ulcerative colitis
ceRNA: Competing endogenous RNA
miRNA: MicroRNA
WGCNA: Weighted gene correlation network analysis
HITS-CLIP: High-throughput sequencing of RNA isolated by crosslinking immunoprecipitation techniques
PPI: Protein-protein interaction
IBD: Inflammatory bowel disease
GEO: Gene Expression Omnibus
GO: Gene Ontology
IL: Interleukin
TNF-*α*: Tumor necrosis factor-alpha
ROC: Receiver operating characteristic
STRING: Search Tool for the Retrieval of Interaction Genes
GS: Gene significance
MM: Module membership
SH3BP5-AS1: SH3BP5 antisense RNA 1
MIR4435-2HG: MIR4435-2 host gene
ENTPD1-AS1: ENTPD1 antisense RNA 1.

## Supplementary Materials

Supplementary Materials Supplementary Table S1: detailed information of the GEO datasets. Supplementary Table S2: baseline characteristics of individuals from the gene expression study. Supplementary Table S3: baseline characteristics of individuals from the miRNA expression study. Supplementary Table S4: module preservation across datasets. Supplementary Table S5: hub lncRNA/gene from 4 modules. Supplementary Table S6: hub miRNA from GSE48959 modules. Supplementary Table S7: Spearman's rank correlation tests between the RNAs of the ceRNA network and the inflammatory cell. Supplementary Table S8: disease evaluation variables are encoded into ordinal variables. Supplementary Figure S1: PPI network of hub genes of the turquoise module. Supplementary Figure S2: abnormal samples filtered by sample clustering analysis.
