# Supplementary material for: The Construction and Comprehensive Analysis of Inflammation-Related ceRNA Networks and Tissue-Infiltrating Immune Cells in Ulcerative Colitis Progression
Source: Biomed Res Int. 2021 Jul 6;2021:6633442. doi: 10.1155/2021/6633442 (PMC8277522; doi:10.1155/2021/6633442)
Supplement: Supplementary Materials — Supplementary Table S1: detailed information of the GEO datasets. Supplementary Table S2: baseline characteristics of individuals from the gene expression study. Supplementary Table S3: baseline characteristics of individuals from the miRNA expression study. Supplementary Table S4: module preservation across datasets. Supplementary Table S5: hub lncRNA/gene from 4 modules. Supplementary Table S6: hub miRNA from GSE48959 modules. Supplementary Table S7: Spearman's rank correlation tests between the RNAs of the ceRNA network and the inflammatory cell. Supplementary Table S8: disease evaluation variables are encoded into ordinal variables. Supplementary Figure S1: PPI network of hub genes of the turquoise module. Supplementary Figure S2: abnormal samples filtered by sample clustering analysis. [file 6633442.f1.docx]

The Construction and Comprehensive Analysis of inflammation-related ceRNA Networks and Tissue-Infiltrating Immune Cells in Ulcerative colitis progression

Jia-Wei Lu^1^, Aimaier Rouzigu^1^, Li-Hong Teng^1^, Wei-Li Liu^2*^

^1^Department of Gastroenterology
Sir Run Run Shaw Hospital, School of Medicine, Zhejiang University
Hangzhou 310016, Zhejiang Province, China

^2^Department of General Practice
Sir Run Run Shaw Hospital, School of Medicine, Zhejiang University
Hangzhou 310016, Zhejiang Province, China

Abstract

Ulcerative colitis (UC) is a common disease with great variability in severity, with a high recurrence rate and heavy disease burden. In recent years, the different biological functions of competing endogenous RNA (ceRNA) networks of long non-coding RNAs (lncRNAs) and microRNAs (miRs) have aroused wide concerns, ceRNA-network of Ulcerative colitis (UC) may have potential research value, these expressed non-coding RNAs may be involved in the molecular basis of inflammation recurrence and progression. This study analyzed 490 colon samples associated with UC from 4 gene expression microarrays from the GEO database and identified gene modules with weighted correlation network analysis (WGCNA). CIBERSORT detected tissue-infiltrating leukocyte profiling by deconvolution of microarray data. LncBase and multiMIR were used to identify lncRNA-miRNA-mRNA interaction. We constructed a ceRNA network which includes 4 lncRNAs (SH3BP5-AS1, MIR4435-2HG, ENTPD1-AS1, AC007750.1), 5 miRNAs(miR-141-3p, miR-191-5p, miR-192-5p, miR-194-5p, miR196-5p) and 52 mRNAs. Those genes involved in interleukin family signals, neutrophil de-granulation, adaptive immunity and cell adhesion pathways. LncRNA MIR4435-2HG is a variable in the decision tree for moderate-to-severe UC diagnostic prediction. Our work identifies a potential regulated inflammation-related lncRNA-miRNA-mRNA regulatory axes. The regulatory axes are dysregulated during the deterioration of UC, suggesting it is a risk factor for UC progression.

1. Introduction

Ulcerative colitis (UC) is a chronic and incurable inflammatory disease, which most often affects the gastrointestinal tract. A recent study also reported that UC prevalence is increasing rapidly worldwide with a high recurrence rate and heavy disease burden(1). Patients with UC need a lifelong course of drugs, which results in high psychological and financial burdens to patients. An emerging biological agent, mainly monoclonal antibodies against cytokines, has been developed, including infliximab and adalimumab (anti-TNF-α agents), golimumab (another anti-TNF-α agent), tofacitinib (a Janus kinase inhibitor), vedolizumab and etrolizumab (integrin antagonists)(2). Nevertheless, up to 30% of patients show no clinical benefit following Biopharmaceutical. It is urgent to deepen the understanding of the pathogenesis of ulcerative colitis progression at the molecular level to determine new therapeutic and disease surveillance strategies. The gene expression network of the disease has been predicted to be sophisticated and fathomable, involving a large variety of characters, such as transcription factors (TFs), microRNAs (miRNAs), long noncoding RNAs (lncRNAs) and protein-coding genes(mRNAs).

One of the regulated axes call competing endogenous RNA network(ceRNA), ceRNA hypothesis was first proposed by Salmena et al. in 2011(3). miRNAs loaded in the RNA-induced silencing complex (RISC) post-transcriptionally regulate protein-coding genes through mRNA cleavage, direct translational repression and/or mRNA destabilization. lncRNAs can compete with mRNAs for miRNA binding by acting as "sponge" molecules in both cell compartments.

The role of ceRNA network has attracted much attention in recent years. Recently, there has been a lot of research and analysis of ceRNA, of which tumor analysis is the most extensive. Many studies have confirmed that lncRNAs are mediated by proliferation, metastasis, drug sensitivity and tumor progression. Recent research expanded its function into the field of non-tumor. Few studies on ceRNAs have focused on IBD-related mechanisms. So far, Nie and Zhao et al. have shown that Lnc-ITSN1-2 promotes Th1/Th17 cell differentiation and CD4+ T cell activation by sponging miR-125a in increased IBD develop inflammatory cytokines of IBD(4). Ye et al. have shown that the dysregulation of circRNA_103516 in PBMCs may participate in IBD through hsa-miR-19b-1-5p sponging (5).

The complex network of LncRNA-miRNA-mRNA regulatory mechanisms is difficult to determine by exploring individual pair interactions. Therefore, high-throughput sequencing of RNA isolated by crosslinking immunoprecipitation techniques (HITS-CLIP) can directly identify multiple targeting sequences in the samples (6). The high-throughput experimental data provide molecular interaction prediction data (7).

This research emphasize establishing a regulatory network rather than analyzing individual genes that focus on a specific molecular interaction. The functions of the characteristic RNAs were investigated by exploring multiple UC-related microarrays from Gene Expression Omnibus (GEO) datasets. We elucidated their possible participation in UC pathogenesis and established ulcerative colitis progression associated ceRNA network. Overall, the effects and potential underlying molecular mechanisms of RNAs on the pathological process of Ulcerative colitis were elaborated through interactions with specific RNAs. We explored an inflammatory-related ceRNA network including 4 lncRNAs, 5 miRNAs and 52 mRNAs. The ceRNA network may involve the interaction among tissue-infiltrating immune cells, including neutrophils, macrophage M0/M1, CD8+T cell and regulatory T cell. The analysis indicated that the ceRNA network might become a potential genetic risk factor for ulcerative colitis.

1. Materials and Methods
   1. Data collection and data processing

The Gene Expression Omnibus (GEO, https://www.ncbi.nlm.nih.gov/geo/) database from the National Center for Biotechnology Information was searched for publicly available studies, and samples that fulfilled the following criteria for analysis: (1) the species of the samples was Homo sapiens; (2) The gene expression data series contained UC colon tissue and normal colon tissue samples; (3) the selected dataset should be over 15 samples; (4) the characteristic of samples from gene expression study contained UC activity assessment. We used five adult human UC microarray data sets from GSE48959, GSE73661, GSE75214, GSE87466 and GSE92415. Among them, GSE48959, GSE73661 and GSE75214 were hybridized to Affymetrix Human Genome Affy Human Gene 1.0ST Genechips (Affy Human Gene 1.0ST, Affymetrix), while GSE87466 and GSE92415 were profiled using U133A Genechips (HG-U133A, Affymetrix), miRNAs array profiling from GSE48959 was performed with Affymetrix Multispecies miRNA-2 Array. By reannotating the mRNA microarray data for lncRNAs. Samples contain gene expression matrix and baseline characteristics of 507 ulcerative colitis samples and 62 normal colon samples, of which 17 colitis samples and 10 normal samples come from miRNA datasets. Microarray data were normalized, the batch effect was assessed and removed by the removeBatchEffect function from the limma R package.

- 1. Established tissue-infiltrating immune cells signature matrix (LM22)

Using CIBERSORT analysis to analyze LM22 abundance in each sample and filter out abnormal data(8), subsequently, we calculated the correlation coefficient between the abundance of cell subset of the samples and the rank variable of the disease status of their represented samples.

- 1. Identify modules of co-expressed genes within gene expression networks

We used weighted gene co-expression network analysis (WGCNA) to identify modules of co-expressed genes within gene expression networks. (9) WGCNA was implemented in R. we performed automatic network construction and module detection with the following major parameters: Signed network, power of 20, maxBlockSize of 20000, minModuleSize of 50, verbose of 3 and mergeCutHeight of 0.25. Module membership (MM) represents the intramodular connectivity of any gene in a given module. A higher absolute value of MM represented a gene has a greater negative or positive correlation with the module eigengenes (MEs). Gene significance (GS) is used to incorporate clinical characteristics into the co-expression network. A higher value of GS indicates the increased biological significance of a gene for a given clinical trait. Hub genes in key modules were identified based on |MM|>0.8 and GS>0.2.

- 1. Weighted gene co-expression network meta-analysis

The reliability of the modules was checked by module preservation analysis. These datasets were independently processed depending on the platform, and the expression of module genes was used as input data to quantify the extent of preservation in each dataset. A statistics method proposed by Langfelder et al. was used to find the extent of module preservation (10). The following thresholds for Zsummary were used: no preservation (Zsummary < 2), weak to moderate evidence of preservation (2 < Zsummary< 10) and strong evidence of module preservation (Zsummary > 10).

- 1. Enrichment analysis and enrichment map of enrichment result

To identify represented pathways in co-expression modules related to UC development, we used ClusterProfiler in the R Bioconductor package (11), significantly enriched GO terms in genes in a module comparing to the background were defined by hypergeometric test and with a threshold of false discovery rate (FDR) less than 0.05. Results were visualized by bar plots created by the ClusterProfiler package. The Enrichment Map results from gene set enrichment analysis created by enrichplot package (R package).

- 1. Protein-protein interaction (PPI) networks

StringAPP constructed A comprehensive human PPI network (12). This study integrates hub genes of differential co-expression module. A PPI network was constructed by mapping the gene expression to the PPI network. We overlapped our gene list with the PPI network, set the confident threshold of 0.7, and removed the non-interacting nodes.

- 1. Construction of a ceRNA Network

Based on the hypothesis, the candidate LncRNAs and mRNAs expression must have the same variation trend, while miRNA should be opposite trend. LncRNA-miRNA interactomes were performed using the LncBase experimental database (13), miRNA-mRNA interactions were obtained from the R package "muitiMIR"(14). The obtained LncRNA-miRNA pairs and miRNA-mRNA pairs were combined to construct a ceRNA network. The Cytoscape software (v3.8.0) was used to visualize the ceRNA networks (15).

- 1. Establish a severity detection for the UC patients using a decision tree

The patient's clinical data were extracted from the clinical data of UC patients in two GEO datasets. 328 UC samples studied in GSE73661 and GSE92415 were included. Outliers were filtered out by choosing an appropriate cut parameter of the height of the tree cut in the dendrogram. In order to make the established prognostic model have better generalization ability, we established a decision tree model using rpart (R package). We train and provide better model results by Caret (R package). After performing 10-fold cross-validations, the cost-complexity parameter (Cp) value in which the test error was minimized was selected as the optimal Cp value. Classification models were evaluated based on the area under the ROC curve (AUC) using the "pROC(16)" software package(R package) and confusion matrix(R package)(17).

1. Results
   1. Pre-processing of the lncRNA/mRNA datasets and construction of weighted gene co-expression networks

The overview of this study was shown in Figure 1A. We screened qualified datasets from GEO and finally, four gene expression datasets were downloaded from GEO(GSE48959(18), GSE73661(19), GSE75214(20), GSE92415(21) ).In summary, 52 normal colon biopsy samples and 438 UC colon biopsy samples were included. (The total patients characteristic is shown in Table S1-2) All of these samples contain an evaluation of disease activity by both endoscopy and symptoms. The evaluation variables are further categorized into ordinal variables. Ordinal variables allow us to order the disease severity in terms of which category has less and which category has more severe represented by the variable. An ordinal variable is ranked by the mayo score from GSE92415, mayo endoscopic score from GSE73661 and disease activity assessment from GSE48959 and GSE75214(Table S8). We identified 15088 common mRNAs and 1866 common lncRNAs from four datasets. Using the WGCNA method, 25 gene modules were constructed. A gene module is considered as a set of co-expressed genes to which the same set of transcription factors binds. Those modules were first validated by assessing their preservation across datasets (Figure 1B and Table S4). Remarkably, the co-expression structure of those modules from GSE92415 can be reproducibly identified in either of three independent expression datasets, especially in blue, dark green, green, grey60, light-yellow, royal blue, tan, turquoise modules. The next step is to illustrate the correlation between gene module and disease severity. We noticed that turquoise modules positively related to the severity of ulcerative colitis among four datasets (Figure 1C), green, blue and tan modules negatively related to the development of ulcerative colitis among four datasets (Figure 1C), the bar plots revealed that the eigengene value of modules correlated with the disease severity (Figure 1D). The above work shows that four modules (turquoise, green, blue and tan) are significantly related to ulcerative colitis severity. Subsequently, we used a relatively high criterion to select hub RNAs on 4 modules (Table S5). Finally, we identified 329 hub mRNAs and 23 hub lncRNAs in the turquoise module, 189 hub mRNAs and 13 hub lncRNAs in the blue module, 76 hub mRNAs and 1 hub lncRNAs in the green module and 31 hub mRNAs and 3 lncRNAs in the tan module.

Figure 1- Pre-processing of the lncRNA/mRNA datasets and construction of weighted gene co-expression networks. (A) Flow chart for an overview of the present analysis. (B) Preservation of GSE92415 network modules in different datasets. The y axis displays the Z-score for each module. Labels beside each module (colored dot) represent the corresponding module in the reference dataset. The x-axis represents the number of genes in the module. Z scores of less than 2 (blue bottom line) imply no evidence for module preservation, while scores exceeding 10 (red line) and exceeding 10 implies strong evidence for module preservation. (C) Module trait relationship: A matrix with the Module-Trait Relationships (MTRs) (correlation coefficients) and corresponding p-values (in brackets) between modules on the y-axis and disease progression traits of four datasets on the x-axis. (D) The relationship between module eigengene and disease severity, the y-axis represents the module eigengene expression value of the sample, and the samples are sorted from mild to severe disease status.

(a)

(b)

(c)

(d)

- 1. Construction of Weighted Gene Co-expression Networks in miRNA dataset.

GSE48959 microarray dataset also contains miRNA expression data. In total, there were 8 normal colon biopsy samples and 16 ulcerative colitis colon biopsy samples. (The total patients characteristic is shown in Table S3), we obtained 1067 common miRNAs from the datasets. Using WGCNA, two modules correlated with ulcerative colitis progression are detected (Figure 2A), of which 11 hub-miRNA of the brown module were upregulated and 20 hub-miRNA of the blue module were downregulated (Table S6), and we noticed that the module eigengene value also correlated with the disease severity (Figure 2B).

Figure 2- miRNA module trait relationship. (A) A matrix with the miRNA module-Trait Relationships (correlation coefficients) and corresponding p-values (in brackets) between modules on the y-axis and disease progression traits of multiple datasets on the x-axis. (B) The relationship between module eigengene and disease severity, the y axis represents the module eigengene expression value of the sample, and the samples are sorted from mild to severe disease status.

(a)

(b)

- 1. GO enrichment and pathway analysis of modules

We performed pathways and Gene Ontology (GO) enrichment analyses of the identified hub gene sets function in modules. The main functions and pathways enriched by the hubs in these modules are shown. The function enrichment of the turquoise module was found to participate in inflammation development (P<0.05, Figure 3A). these genes were enriched for the Gene Ontology categories related to leukocyte cell adhesion and proliferation, as well as neutrophils activation and de-granulation, also stimulated to cytokines release and cytokines receptor activity, pathway enrichment demonstrated that hub genes involved in inflammation-mediated by extracellular matrix (ECM) organization, leukocyte-cell adhesion, immune response-activating cell receptor signal and cytokine receptor activity, Etc. (P<0.05, Figure 3B) The blue module showed a significant decrease with a small molecular catabolic process, microvillus organization, steroid hormone receptor activity. The green module showed a significant decrease with cell-cell junction, organic anion transport, endocytic vesicle membrane formation. The tan modules showed a significant decrease in ion channel/transporter activity, lipid metabolism and biological oxidation (P<0.05, Figure 3A). The results may indicate that mitochondrial function and various metabolism were dysfunctional during the progression. Tissue structure formation and bowel barrier were also damaged, preventing mucosal wound healing and disrupting the mucus's protective functions layer and contributing to recurrent and deterioration of inflammatory stimulation.

Figure 3-GO enrichment and Reactome pathway analysis for dysregulated gene modules. (A) Bar charts showing the top 5 GO terms for biological process (BP), cellular component (CC), molecular function (MF) in four modules (P < 0.05). (B) Pathway crosstalk among gene-enriched pathways of the turquoise module. Nodes represent pathways, and edges represent crosstalk between pathways.

(a)

(b)

- 1. Construction of an inflammation-related ceRNA network

We identified the turquoise module as significantly upregulated and mainly related to the inflammation process, then we constructed the PPI network-associated turquoise module's mRNAs (Figure S1). Based on ceRNA hypothesize, we selected the negative interactomes from the ceRNA network to construct regulatory axes. 23 upregulated hub lncRNAs from the turquoise lncRNA/mRNA module, 20 downregulated miRNAs from the blue miRNA module and 195 upregulated mRNAs in the PPI network from the turquoise lncRNA/mRNA module were first screened. The negatively correlated miRNA-mRNA pairs in the ceRNA network were detected through synthesizing miRDB, miRTarBase, TargetScan validation, lncRNA-miRNA pairs were detected by DIANA lncBase v3 database. (see Method section 2.7). Finally, a network consisting of 4 lncRNAs, 5 miRNAs and 52 mRNAs was constructed and visualized (Table 1 and Figure 4A). A Diagram of the correlation relationships between lncRNAs, miRNAs and mRNAs revealed that all miRNAs expressions significantly correlated with their targeted lncRNAs and targeted mRNAs in GSE48959 datasets (Figure 4B).

Table 1 ceRNA regulation network of lncRNAs, miRNAs and mRNAs in UC

| lncRNA | miRNA | mRNA |
| --- | --- | --- |
| AC007750.1 | hsa-miR-191-5p | ANGPTL2, ARHGDIB, CD79A, COL1A2, CSGALNACT1, CSGALNACT2, DOK3, FYN, HRH2, ICAM1  , INPP5D, ITGAM, MAP3K3, NCF2, OSMR, P2RY8, PIK3CD, PIP4K2A, PLAU, SOCS3 |
| SH3BP5-AS1 | hsa-miR-192-5p  hsa-miR-194-5p  hsa-miR-196a-5p | FBN1, LIMS1, MCAM, MSN, NOD2, PLAU, SEMA4D, THBD, TNFSF13B, FSCN1, LOXL2, COL1A2, CSGALNACT2, SPARC, CSGALNACT2, CXCR4, CYTIP, IGFBP5, MAP3K3, MSN, RAC2, ROBO1, SLAMF1, SLC2A3, SOCS3, SRGN, TGFBI, TNC, WIPF1, ATP11A, FSCN1, COL1A2, CSGALNACT2, FBN1, THBS2, FSTL1, RASGRP1, RASSF5 |
| MIR4435-2HG | hsa-miR-196a-5p | ATP11A, FSCN1, COL1A2, CSGALNACT2, FBN1, THBS2, FSTL1, RASGRP1, RASSF5 |
| ENTPD1-AS1 | hsa-miR-194-5p  hsa-miR-196a-5p | MAP4K4, QKI, MMP2, MMP9, STAT4, ATP11A, FSCN1, COL1A2, CSGALNACT2, FBN1, THBS2, FSTL1, RASGRP1, RASSF5 |

Figure 4- Construction and Correlation analysis of ceRNA regulatory network. (A) lncRNA-miRNA-mRNA interacted network. The squares represent the downregulated miRNA, the rhombus represents the upregulated lncRNA, and the circles represent the upregulated mRNA. (B) The data were visualized by heatmap, with a positive correlation in red and those negative in blue. The graduated color represents the correlation coefficient (ranging from 0, pale colors, to ±1, deep colors). Pearson's correlation test calculated the correlation coefficient.

(a)

(b)

- 1. The Correlation of disease progression and the abundance of Tissue-infiltrating immune cells

We discuss the relative abundance of different tissue-infiltrating immune cell types analyzed by CIBERSORT. The results are listed in order of severity for disease (Figure 5A). As the disease progressed, immune cell composition changes as an evolution of the disease. For better understanding, we analyze the Spearman's Rank Correlations between the abundance of tissue-infiltrating immune cells and severity for disease (Figure 5B), the abundance of six subsets of immune cells (neutrophils, resting NK cell, Macrophage M0/M1, activated dendritic cell and activated mast cell) were correlated positively and significantly with the progression. In comparison, five subsets (regulatory T cell, CD8+ T cell, activated NK cell, Macrophage M2 and resting mast cell) were significantly negatively correlated. In summary, the results showed that the deterioration of UC is correlated with infiltration of the pro-inflammatory cell.

Figure 5-The correlation of disease progression and changes in tissue-infiltrating immune cells (A) Enrichment scores for 22 tissue-infiltrating immune cells subpopulations on four gene expression datasets based on deconvolution by CIBERSORT. The results are listed in order of disease severity assessment (GSE73661, mayo endoscopic score (MES): 0-3; GSE48959 and GSE75214, disease activity assessment: Normal, inactive UC, active UC; GSE92415, mayo score: 0-12). (B) A heat map of the Spearman rank correlation coefficient between the proportion of 19 tissue-infiltrating immune cells and disease characteristics on four gene expression datasets. Asterisks denote significant correlations after P value corrections (P < 0.05).

(a)

(b)

- 1. the relationship between ceRNA network and inflammation

The enrichment showed that the ceRNA network genes mainly enriched in interleukin family signals (especially in IL-4 and IL-13 signaling), neutrophil de-granulation, adaptive immunity, cell surface interaction at the vascular wall and integrin-cell surface interactions (Figure 6A). We also assessed the correlation between these expressions of RNAs and tissue-infiltrating immune cells, and the correlation analysis showed that mRNAs, miRNAs and lncRNAs were moderately and highly associated with most of the UC-related infiltrating immune cells in respective datasets, in particular, neutrophil, macrophage M1, macrophage M0, CD8+T cell and regulatory T cell (Figure 6B-C).

Figure 6- Correlation between the gene of the ceRNA network and inflammatory pathway. (A) Heatmap represents an association matrix of mRNAs in the ceRNA network and Reactome pathway terms. (B) Correlation analysis between the abundance of tissue-infiltrating immune cells and the expression of lncRNAs/mRNAs in respective datasets. Asterisks denote significant correlations after P value corrections (P < 0.05). (C) Correlation analysis between the abundance of tissue-infiltrating immune cells and the expression of miRNAs in respective datasets. Asterisks denote significant correlations after P value corrections (P < 0.05).

(a)

(b)

(c)

- 1. Establish a Severity detection for the UC Patients using a decision tree

We established a severity detection model for detecting disease status based on the gene expression of microarray sample and verified the robustness of our model predictions and inference to assess their suitability for disease surveillance. Based on the current treatment options for UC, the therapy plan of moderate-to-severe status is quite similar (22) (moderate-to-severe ulcerative colitis defined as a total Mayo score of 6 to 12 points and Mayo endoscopic score of 2 to 3). Moreover, patients with inactive and mild UC are considered to be in remission status. Therefore, we classified clinical phenotypes into inactive-to-mild or moderate-severe ulcerative colitis, and the normal control group was excluded from this model, then the expressions of lncRNA in the ceRNA network were treated as diagnosis variables. The 328 UC samples from GSE73661 and GSE92415 were first included. To avoid overfitting, we also detected and filtered out the outlier samples (Figure S2). In total, 249 moderate-to-severe patients (80.3%) among the 310 colon samples were treated as a training set. GSE75214, GSE87466(23) were regarded as the external validation set. Decision tree model were fit by machine learning method for acquire the optimize complexity parameter value(Figure 7A), Overall, lncRNA MIR4435-2HG were selected as the key features, with a sensitivity of 83.9%,specificity of 83.6%, balanced accuracy of 83.8%(Figure 7B), There was also a good concordance with validation sets GSE75214 and GSE87466 judged by the model(GSE75214: sensitivity : 93.2%, specificity : 91.3%, balanced accuracy : 92.3% ; GSE87466: sensitivity : 82.6%, specificity : 90.5%, balanced accuracy : 86.6%,respectively). These are under the ROC in the training set, validation set GSE75214 and validation set GSE87466 was 0.84(95% CI 0.79-0.89), 0.92(95% CI 0.86-0.99), and 0.87(95% CI 0.79-0.94) respectively, which indicates a good ability to distinguish Moderate-to-Severe UC(Figure 7E). The results reveal that this model may provide a gene expression-based disease surveillance of UC.

Figure 7- Establish a Severity detection for the UC Patients using a decision tree. (A) Decision tree. (The normalized MIR4435-2HG expression values are shown, all gene expressions were standardized during the calculation.) (B) Confusion matrix for the training dataset. (C) Confusion matrix for the validation set GSE75214. (D) Confusion matrix for the validation set GSE87466. (E) ROC curve of the model for the training set and two validation sets. The vertical axis represents sensitivity, and the horizontal axis represents specificity.

(a)

(b)

(c)

(d)

(e)

1. Discussion

In this article, we found that interleukin family-related pathways, focal adhesion, extracellular matrix adhesion enriched in ceRNA networks, those are known to play an essential role in ulcerative colitis in the literature review (24-26), Interleukin-13 and Interleukin-4 are produced by CD4+ Th2 cells, mediates UC through the shared type II interleukin-4 receptor, as it co-operated with tumor necrosis factor-alpha (TNF-α) to regulate the expression of genes responsible for the development of tight junction entero-epithelial cells. (27) Bram Verstockt et al. suggested that IL-13Rα2 on epithelial cells contributes to IBD pathology by negatively regulating goblet cell recovery and epithelial restoration after injury (28). Blocking IL-13Rα2 might be a promising target for the recovery of the bowel epithelial barrier in IBD, safety data of drugs targeting the IL-13 and IL-4 pathway are reassuring, and there were also a small number of studies that demonstrated protective effects of IL-13 and IL-4 pathways inhibition of signaling by both IL-13 and IL-4, through KO of IL-4Rα reduced the proliferation of malignant cells and increased apoptosis in a mouse model of colorectal cancer. However, several lines of evidence challenge this safety, Braddock et al. has proved that IL-13 and IL-4 may have roles in the development of colorectal cancers (29). Therefore, more research needed to eliminate the current disarray in the literature.

We also discovered that the abundance of inflammatory cells in m0/m1 cells, activated mast cells, neutrophils and cd4t cells were significantly positively correlated with the disease progression. M0/M1 cells are highly related to the deterioration of the disease. Many articles have shown that m1 cells in inflammatory bowel disease are the main cell type that releases inflammatory factors (30-32). It is known that LPS and IFN-γ can activate M1 macrophages via the nuclear factor kappa-B (NF-κB) signaling pathway, producing the pro-inflammatory factors IL-1β, TNF-α, IL-6, IL-23, reactive oxygen species, nitric oxide (NO) and inducible nitric oxide synthase (iNOS)(33). Thus, M1 macrophages are predominant in the early stage of inflammation. The potential function of mast cells to IBD has been demonstrated in experimental studies. The mast cell mediators involved in IBD pathogenesis include TNF-α, IL-6, histamine, prostaglandins and leukotrienes. Mercé Albert-Bayo reviews conclude that mast cells play a role in intestinal permeability, initiation and maintenance of inflammatory processes with ensuing tissue remodeling and neuropathological stress (34). Combined with the functional enrichment of whose three down-regulated modules, the tissue lipid/small molecular metabolism level was decreased, tissue and cell structure were also damaged. It was exacerbating the defective gut barrier in ulcerative colitis patients. A pro-inflammatory cytokine loop overrides anti-inflammatory signals and causes chronic intestinal inflammation.

Based on the results, a potential ceRNA regulatory axis that well is fitted the ceRNA pattern, the lncRNA MIR4435-2HG, is highly enriched with several cytokine pathways. The massive report indicated that LncRNA MIR4435-2HG contributes to colorectal cancer development and predicts poor prognosis. MIR4435-2HG was identified as a miRNA sponge for TGF-β1 and thus activated TGF-β signaling, which indicates that MIR4435-2HG may also play an inflammation-mediated role. Dong X et Al. MIR4435-2HG was highly expressed in CRC tissue compared to normal tissues, displaying poor prognosis (35, 36). Overall, the literature review suggested that MIR4435-2HG knockdown could suppress CRC cell proliferation, invasion and migration. MIR4435-2HG may be a key mediator of both inflammatory processes and colorectal cancer generation. In addition to lncRNAs, miRNAs, as a widely discussing non-coding RNA, are critical in the ceRNA hypothesis. We found a complex regulatory network for the above 5 miRNAs, and the miRNAs have extensively been studied. Mechanisms involving miRNAs have been shown to take part in various autoimmune diseases, including IBD. Wu et al. first evaluated the abnormal expression of miRNA miR-192 in the intestinal tissue of UC patients, decreases TNF-α induced MIP-2-α expression, also shown to be pro-fibrotic (37), miR-192 can inhibit the expression of NOD2, inhibiting innate immune system activation via the NF-κB pathway, and inhibit interleukin-8 and CXCL3 messenger RNA expression (38), indicated that mir-192 might protect colon tissue from the damage by participating inhibition of innate immune signaling. Another miRNA, mir-194-5p. Lin El et Al. was confirmed the specific differential expression of miR-194 in ulcerative colitis (39), miR-194 has more attention on several autoimmune diseases, Tian et al. shows that overexpression of miR-194 attenuated the release of pro-inflammatory cytokine TNF-α in PA-activated monocyte THP-1 in rheumatoid arthritis (40). Another downstream miRNA mir-196a is associated with various diseases, including Rheumatoid arthritis (41), colorectal cancer (42-44). In the review about IBD, Ranjha et al. found that miR-196a-2 was also negatively associated with UC (45). However, Brest et al. found that the expression of miR-196 is increased in colon epithelial cells exhibiting Crohn disease-associated inflammation due to downregulate the protective immunity-related GTPase family M protein (IRGM), which affected the function of autophagy (46). The pathogenesis of UC and Crohn's disease is considerably distinct from each other. The dysbiosis, impairment of the epithelial barrier via disruption of tight junctions are strongly implicated in the pathogenesis of UC (22).

Studies evaluating biological treatments in patients with severe disease and inadequate response to conventional therapies were remarkably cost-effective. We have attempted to use multiple methods, including the widely used regression model, to establish a diagnostic prediction model, but high-throughput microarray data may not be applicable for constructing the regression model due to big noise and high dimensions. This study tested the performance of supervised machine learning algorithms: Decision Forest Regression (DFR), disease modeling with DFR has several distinct advantages, the approach, which automates the feature selection, efficiently selected the few critical features from the false signal, and the model has a simple structure based on one optimal attribute of each split dot. Our decision tree confirms that lncRNA MIR4435-2HG variables are good predictors. The goodness of fit assessment suggested that our model fit well with the data. The accuracy of the decision tree is also relatively high.

This article addresses several limitations in this literature. First, it is unfeasible for a current database to include all RNA-RNA interaction information. Hence, some of the potential interactomes may not be included in our network, and some critical regulatory RNAs might be lost of the annotations. Second, despite the progress in developing the ceRNA network, the hypothesis still lacks conclusive evidence. Most of the active miRNA were not readily affected by ceRNA. The natural conditions in cells are difficult to control, and it is easy to overexpress genes artificially, which cannot mimic the normal ceRNA effects in the body.

Moreover, there are not many prediction tools currently available. Third, most of the miRNA-mRNA predicted datasets are based on 3'UTR sequences, which have certain limitations. An accumulating amount of evidence indicates lncRNAs play an important role in biological functions through multiple regulation levels, which involve transcriptional, post-transcriptional and epigenetic regulation.

1. Conclusions

Our work combined four microarray data from the GEO database and identified potential inflammatory gene-related ceRNA network regulatory axes dysregulated during UC progression. We speculate that the lncRNA-miRNA-mRNA regulatory axes may depend on the interaction among tissue-infiltrating immune cells, including neutrophil, macrophage M0/M1, CD8+T cell and regulatory T cell. These RNAs play a crucial role in inflammatory stimulation and its abnormal behavior in UC, as reported. The network and its potential function obtained from the bioinformatics analysis can be examined by future experimental studies.

Abbreviations

UC: Ulcerative colitis

ceRNA: competing endogenous RNA

miRNA: microRNA

WGCNA: Weighted gene correlation network analysis

HITS-CLIP: High-throughput sequencing of RNA isolated by crosslinking immunoprecipitation techniques

PPI: Protein-protein interaction

IBD: Inflammatory bowel disease

GEO: Gene Expression Omnibus

GO: Gene Ontology

IL: interleukin

TNF-α: Tumor necrosis factor-alpha

ROC: Receiver Operating Characteristic

STRING: Search Tool for The Retrieval of Interaction Genes

GS: Gene Significance

MM: Module Membership

Gene Abbreviation

SH3BP5-AS1: SH3BP5 antisense RNA 1

MIR4435-2HG: MIR4435-2 Host Gene

ENTPD1-AS1: ENTPD1 Antisense RNA 1

Data Availability

The data supporting this article are from previously reported studies and datasets, which have been cited. The processed data and code are available from the corresponding authors upon request.

Conflicts of Interest

The authors declare that they have no competing interests.

Authors' contributions

All authors contributed to the work presented in this paper. Conceptualization, JW. Lu, Aimaier Rouzigu, and Li-Hong Teng; GEO resources, analysis, visualization and validation JW. Lu; writing—original draft preparation, WL. Liu and JW. Lu.; writing—editing, WL. Liu, supervision, WL. Liu; project administration, WL. Liu, all authors have read and agreed to the published version of the manuscript.

Funding

No funding was supported from any program or foundation.

1. Supplementary Materials

Supplementary Materials Supplementary Tables Supplementary Table S1: Detailed information of the GEO datasets Supplementary Table S2: Baseline characteristics of individuals from gene expression study Supplementary Table S3: Baseline characteristics of individuals from miRNA expression study Supplementary Table S4: Module preservation across datasets Supplementary Table S5: Hub lncRNA/gene from 4 modules Supplementary Table S6: Hub miRNA from GSE48959 modules Supplementary Table S7: Spearman's Rank Correlation tests between the RNAs of ceRNA network and the inflammatory cell Supplementary Table S8: Disease evaluation variables are encoded into ordinal variables Supplementary Figures Supplementary Figure S1: PPI network of hub genes of turquoise module Supplementary Figure S2: Abnormal samples filter by sample clustering analysis

References

1. Jean L, Audrey M, Beauchemin C, Consortium O. Economic Evaluations of Treatments for Inflammatory Bowel Diseases: A Literature Review. Can J Gastroenterol Hepatol. 2018; 2018:7439730.

2. Park SC, Jeen YT. Current and emerging biologics for ulcerative colitis. Gut Liver. 2015; 9(1): 18-27.

3. Salmena L, Poliseno L, Tay Y, Kats L, Pandolfi PP. A ce RNA hypothesis: the Rosetta Stone of a hidden RNA language? Cell. 2011; 146(3): 353-8.

4. Nie J, Zhao Q. Lnc-ITSN1-2, Derived From RNA Sequencing, Correlates With Increased Disease Risk, Activity and Promotes CD4(+) T Cell Activation, Proliferation and Th1/Th17 Cell Differentiation by Serving as a ce RNA for IL-23R via Sponging mi R-125a in Inflammatory Bowel Disease. Front Immunol. 2020; 11: 852.

5. Ye YL, Yin J, Hu T, Zhang LP, Wu LY, Pang Z. Increased circulating circular RNA_103516 is a novel biomarker for inflammatory bowel disease in adult patients. World J Gastroenterol. 2019; 25 (41): 6273-88.

6. Reuter JA, Spacek DV, Snyder MP. High-throughput sequencing technologies. Mol Cell. 2015; 58(4): 586-97.

7. Chen X, Sun Y, Cai R, Wang G, Shu X, Pang W. Long noncoding RNA: multiple players in gene expression. BMB Rep. 2018; 51(6): 280-9.

8. Chen B, Khodadoust MS, Liu CL, Newman AM, Alizadeh AA. Profiling Tumor Infiltrating Immune Cells with CIBERSORT. Methods Mol Biol. 2018; 1711: 243-59.

9. Langfelder P, Horvath S. WGCNA: an R package for weighted correlation network analysis. BMC Bioinformatics. 2008; 9: 559.

10. Langfelder P, Luo R, Oldham MC, Horvath S. Is my network module preserved and reproducible? PLoS Comput Biol. 2011; 7(1): e1001057.

11. Yu G, Wang LG, Han Y, He QY. cluster Profiler: an R package for comparing biological themes among gene clusters. OMICS. 2012; 16(5): 284-7.

12. Doncheva NT, Morris JH, Gorodkin J, Jensen LJ. Cytoscape String App: Network Analysis and Visualization of Proteomics Data. J Proteome Res. 2019; 18(2): 623-32.

13. Karagkouni D, Paraskevopoulou MD, Tastsoglou S, Skoufos G, Karavangeli A, Pierros V, et al. DIANA-Lnc Base v3: indexing experimentally supported mi RNA targets on non-coding transcripts. Nucleic Acids Res. 2020; 48 (D1): D101-D10.

14. Ru Y, Kechris KJ, Tabakoff B, Hoffman P, Radcliffe RA, Bowler R, et al. The multi MiR R package and database: integration of micro RNA-target interactions along with their disease and drug associations. Nucleic Acids Res. 2014; 42 (17): e133.

15. Shannon P, Markiel A, Ozier O, Baliga NS, Wang JT, Ramage D, et al. Cytoscape: a software environment for integrated models of biomolecular interaction networks. Genome Res. 2003; 13 (11): 2498-504.

16. Engebretsen S, Bohlin J. Statistical predictions with glmnet. Clin Epigenetics. 2019; 11(1): 123.

17. Luo W, Brouwer C. Pathview: an R/Bioconductor package for pathway-based data integration and visualization. Bioinformatics. 2013; 29 (14): 1830-1.

18. Van Der Goten J, Vanhove W, Lemaire K, et al. Integrated mi RNA and mRNA expression profiling in inflamed colon of patients with ulcerative colitis [J]. PLoS One, 2014, 9(12): e116117.

19. Arijs I, De Hertogh G, Lemmens B, Van Lommel L, de Bruyn M, Vanhove W, et al. Effect of vedolizumab (anti-alpha 4beta7-integrin) therapy on histological healing and mucosal gene expression in patients with UC. Gut. 2018; 67(1): 43-52.

20. Vancamelbeke M, Vanuytsel T, Farre R, Verstockt S, Ferrante M, Van Assche G, et al. Genetic and Transcriptomic Bases of Intestinal Epithelial Barrier Dysfunction in Inflammatory Bowel Disease. Inflamm Bowel Dis. 2017; 23 (10): 1718-29.

21. Li K, Telesco S, Rutgeerts P, Sandborn W, Marano C, Ma K, Johanns J, Zhang H, Strauss R, Reinisch W, Colombel J, Brodmerkel C, et al. Characterization of molecular response to Golimumab in Ulcerative Colitis by mucosal biopsy mRNA expression profiling: results from PURSUIT-SC induction study, accession GSE92415)

22. Yeshi K, Ruscher R, Hunter L, Daly NL, Loukas A, Wangchuk P. Revisiting Inflammatory Bowel Disease: Pathology, Treatments, Challenges and Emerging Therapeutics Including Drug Leads from Natural Products. J Clin Med. 2020; 9(5).

23. Li K, Strauss R, Ouahed J, Chan D, Telesco SE, Shouval DS, et al. Molecular Comparison of Adult and Pediatric Ulcerative Colitis Indicates Broad Similarity of Molecular Pathways in Disease Tissue. J Pediatr Gastroenterol Nutr. 2018; 67(1): 45-52.

24. Christophi GP, Rong R, Holtzapple PG, Massa PT, Landas SK. Immune markers and differential signaling networks in ulcerative colitis and Crohn's disease. Inflamm Bowel Dis. 2012; 18 (12): 2342-56.

25. Giuffrida P, Caprioli F, Facciotti F, Di Sabatino A. The role of interleukin-13 in chronic inflammatory intestinal disorders. Autoimmun Rev. 2019; 18(5): 549-55.

26. Bamias G, Kaltsa G, Ladas SD. Cytokines in the pathogenesis of ulcerative colitis. Discov Med. 2011; 11 (60): 459-67.

27. Heller F, Fromm A, Gitter AH, Mankertz J, Schulzke JD. Epithelial apoptosis is a prominent feature of the epithelial barrier disturbance in intestinal inflammation: effect of pro-inflammatory interleukin-13 on epithelial cell function. Mucosal Immunol. 2008; 1 Suppl 1: S58-61.

28. Verstockt B, Perrier C, De Hertogh G, Cremer J, Creyns B, Van Assche G, et al. Effects of Epithelial IL-13Ralpha2 Expression in Inflammatory Bowel Disease. Front Immunol. 2018; 9: 2983.

29. Braddock M, Hanania NA, Sharafkhaneh A, Colice G, Carlsson M. Potential Risks Related to Modulating Interleukin-13 and Interleukin-4 Signalling: A Systematic Review. Drug Saf. 2018; 41(5): 489-509.

30. Seyedizade SS, Afshari K, Bayat S, Rahmani F, Momtaz S, Rezaei N, et al. Current Status of M1 and M2 Macrophages Pathway as Drug Targets for Inflammatory Bowel Disease. Arch Immunol Ther Exp (Warsz). 2020; 68(2): 10.

31. Liu H, Dasgupta S, Fu Y, Bailey B, Roy C, Lightcap E, et al. Subsets of mononuclear phagocytes are enriched in the inflamed colons of patients with IBD. BMC Immunol. 2019; 20(1): 42.

32. Lissner D, Schumann M, Batra A, Kredel LI, Kuhl AA, Erben U, et al. Monocyte and M1 Macrophage-induced Barrier Defect Contributes to Chronic Intestinal Inflammation in IBD. Inflamm Bowel Dis. 2015; 21(6): 1297-305.

33. Siveen KS, Kuttan G. Role of macrophages in tumour progression. Immunol Lett. 2009; 123(2): 97-102.

34. Albert-Bayo M, Paracuellos I, Gonzalez-Castro AM, Rodriguez-Urrutia A, Rodriguez-Lagunas MJ, Alonso-Cotoner C, et al. Intestinal Mucosal Mast Cells: Key Modulators of Barrier Function and Homeostasis. Cells. 2019; 8(2).

35. Dong X, Yang Z, Yang H, Li D, Qiu X. Long Non-coding RNA MIR4435-2HG Promotes Colorectal Cancer Proliferation and Metastasis Through mi R-206/YAP1 Axis. Front Oncol. 2020; 10: 160.

36. Shen MY, Zhou GR, Z YZ. Lnc RNA MIR4435-2HG contributes into colorectal cancer development and predicts poor prognosis. Eur Rev Med Pharmacol Sci. 2020; 24(4): 1771-7.

37. Wu F, Zikusoka M, Trindade A, Dassopoulos T, Harris ML, Bayless TM, et al. Micro RNAs are differentially expressed in ulcerative colitis and alter expression of macrophage inflammatory peptide-2 alpha. Gastroenterology. 2008; 135(5): 1624-35 e24.

38. Chuang AY, Chuang JC, Zhai Z, Wu F, Kwon JH. NOD2 expression is regulated by micro RNAs in colonic epithelial HCT116 cells. Inflamm Bowel Dis. 2014; 20(1): 126-35.

39. Lin J, Zhang X, Zhao Z, Welker NC, Li Y, Liu Y, et al. Novel Micro RNA Signature to Differentiate Ulcerative Colitis from Crohn Disease: A Genome-Wide Study Using Next Generation Sequencing. Microrna. 2016; 5(3): 222-9.

40. Tian H, Liu C, Zou X, Wu W, Zhang C, Yuan D. MiRNA-194 Regulates Palmitic Acid-Induced Toll-Like Receptor 4 Inflammatory Responses in THP-1 Cells. Nutrients. 2015; 7(5): 3483-96.

41. Xiao Y, Liu H, Chen L, Wang Y, Yao X, Jiang X. Association of micro RNAs genes polymorphisms with arthritis: a systematic review and meta-analysis. Biosci Rep. 2019; 39(7).

42. Xin H, Wang C, Liu Z. mi R-196a-5p promotes metastasis of colorectal cancer via targeting Ikappa Balpha. BMC Cancer. 2019; 19(1): 30.

43. Ye Y, Yang S, Han Y, Sun J, Xv L, Wu L, et al. Linc 00472 suppresses proliferation and promotes apoptosis through elevating PDCD4 expression by sponging mi R-196a in colorectal cancer. Aging (Albany NY). 2018; 10(6): 1523-33.

44. Chen X, Du P, She J, Cao L, Li Y, Xia H. Loss of ZG16 is regulated by mi R-196a and contributes to stemness and progression of colorectal cancer. Oncotarget. 2016; 7(52): 86695-703.

45. Ranjha R, Meena NK, Singh A, Ahuja V, Paul J. Association of mi R-196a-2 and mi R-499 variants with ulcerative colitis and their correlation with expression of respective mi RNAs. PLoS One. 2017; 12(3): e0173447.

46. Brest P, Lapaquette P, Mograbi B, Darfeuille-Michaud A, Hofman P. Risk predisposition for Crohn disease: a "menage a trois" combining IRGM allele, mi RNA and xenophagy. Autophagy. 2011; 7(7): 786-7.
